# Influence of Small-Scale Irrigation on Livelihoods of Rural Farm Households in the Case of Legehida District, Ethiopia

**DOI:** 10.1155/2024/9982796

**Published:** 2024-05-23

**Authors:** Awol Hussen, Arebu Hussen

**Affiliations:** ^1^Department of Agricultural Economics, Wollo University, Dessie, Ethiopia; ^2^Department of Plant Science, Mekdela Amba University, Tuluawlia, Ethiopia

## Abstract

Irrigation development, particularly small-scale irrigation, is one of the most important projects for improving agricultural productivity in a country's rural communities. The extent to which small-scale irrigation has improved household livelihoods in Ethiopia's rural areas is not widely recognized. As a result, research on the influence of small-scale irrigation on farmers' livelihoods in the Legehida district will be sought. The study took a “with” and “without” strategy, comparing farmers who used irrigation against those who did not. For analysis, both quantitative and qualitative data were employed. The survey's respondents were chosen using a random sample approach from both irrigation users and nonuser households. Quantitative data for the study were collected from randomly selected 241 farm households, of which 113 were users and 128 were nonusers, using a semistructured questionnaire. Accordingly, the propensity score matching model was employed to examine the impacts of small-scale irrigation on farmers' livelihoods. The logit model result indicates that cultivated land size, off-farm income, education level, family size, dependency ratio, total livestock unit, and distance to the nearest agricultural extension office/FTC are determinant factors in determining whether to practice irrigation when other factors remain constant. The impact of irrigation on a household's income and food security (in terms of daily calorie intake) was evaluated using a propensity score matching model. The result shows that a positive and significant impact on farmers who use small-scale irrigation has increased the daily calorie intake and annual income of households by 244.162 kilocalories and 5234.258 ETB, respectively, as compared to nonirrigation users. This shows that households that participate in small-scale irrigation activities have a higher annual income and food security status than comparable groups. In general, the study recommends that to reduce food insecurity and the socioeconomic problems of rural households, irrigation farming is one of the viable solutions; therefore, the government and nongovernmental organizations should extensively focus on the enhancement of small-scale irrigation infrastructure, policies, strategies, and extension services to increase productivity, income, and livelihood improvement in rural households.

## 1. Introduction

### 1.1. Background of the Study

Ethiopia is one of the world's poorest countries, with around 29.2% of its people living below the poverty line [1]. The majority of Ethiopians reside in rural regions, where smallholder farmers generate around 95% of agricultural products [2]. Agriculture is the backbone of the Ethiopian economy, employing around 80% of the people directly or indirectly. This suggests that agriculture is the leading contributor to GDP. Agriculture, for example, supplied 40% of national GDP, 80% of employment, 70% of raw material supply, 28% of government tax income, and 85% of export profits in 2011. However, it is largely based on subsistence farming due to small and fragmented landholdings, reliance on natural factors of production, environmental degradation, population growth, limited access to new agricultural technologies, traditional methods of cultivation, and a lack of institutional support services [3].

To address the issue of subsistence farming, economic actors created a national strategic plan in 1991 called Agricultural Development Led Industrialization (ADLI), which focuses on irrigation, cooperative societies, and agricultural technologies to meet food demand and bring socioeconomic development to the country. As they lack agriculture insurance and are less vulnerable to economic shocks, low-income rural families rely heavily on government subsidies to reduce potential losses [4]. One of the policies outlined in this approach is small-scale irrigation development. The success of Asian countries such as China in accommodating a growing population, achieving rapid economic growth, and increasing employment through irrigated agriculture in the 1960s and 1970s has prompted the Ethiopian government to prioritize the development of irrigation schemes [5].

According to Bao and Huang [6], there is a greater likelihood of acquiring loans from the FinTech business for borrowers who reside in locations where there were more COVID cases during the epidemic, have low incomes, and are unemployed. Based on this, the federal and provincial governments, in collaboration with other international and local NGOs, have considerably aided rural farmers in participating in and using irrigated farming. As a result, irrigated acreage, irrigation production, and the number of irrigation farmers in the nation rose by up to 80% between 1990 and 2010 [7].

Irrigation is one of the agricultural methods that is described as the man-made application of water to ensure double cropping and a consistent supply of water in locations where rainfall is inconsistent [8]. Small-scale irrigation is another key intervention to boost agricultural productivity in rural areas of a country. This assists farmers in overcoming rainfall constraints by providing a steady supply of water for agriculture and livestock production [9]. Using small-scale irrigation is crucial to increasing production during times of low rainfall. Through higher incomes, food security, meeting social needs, and reducing poverty, it can help the rural population's overall quality of life [10, 11]. Irrigation development has been proposed as a crucial technique for increasing agricultural production and stimulating economic growth [12]. Irrigation in Ethiopia helps boost farmer income, household resilience, and buffer livelihoods against shocks and stresses by producing higher-value crops for market and harvesting more than once a year. As a result, they were able to accumulate assets, purchase additional food and nonfood household items, educate their children, and reinvest in improving their output by purchasing farm supplies or cattle. However, advantages are dispersed relatively unevenly across families [13]. Large-scale irrigation programs and other associated technologies are well recognized in Ethiopia, and the country's governments actively promote them. Similarly, in recent years, because small-scale irrigation systems are financially viable, investment cost recovery operation, maintenance costs, and the ability to replicate investments, Ethiopia's irrigation program has shifted from large-scale irrigation to small-scale irrigation [14]. It is feasible to completely understand the heterogeneity impacted by COVID-19 policies since rural households' income levels have a significant influence on the degree and intensity of agricultural intensification and operation [4]. Regional states and nongovernmental organizations (NGOs) are pushing for the construction of small-scale irrigation schemes by the country's development policy to boost and stabilize food production in the country. Small-scale irrigation development has been acknowledged as a key strategy for stimulating economic growth and household rural development.

### 1.2. Statement of the Problem

Irrigation boosts agricultural productivity by improving crop yields and allowing farmers to increase cropping intensity and switch to high-value crops [15]. Similarly, according to Lipton et al. [16], irrigated agriculture can reduce poverty by increasing production and income, as well as lowering food prices, which helps very poor households meet basic needs by improving overall economic welfare, protecting against crop loss due to insufficient rainwater supplies, encouraging greater use of yield-enhancing farm inputs, and creating additional employment, all of which allow people to move out of poverty.

Irrigation development, particularly small-scale irrigation, is one of the most important projects for improving agricultural productivity in a country's rural communities. It assists poor households in overcoming rainfall shortages by providing optimal water for irrigation agriculture and livestock, strengthening the foundation for sustainable agriculture, increasing food security in poor communities through irrigated agriculture, and contributing to human nutrition improvement [9]. According to MoARD [2], the importance of irrigation development, particularly at the smallholder level, is critical for increasing productivity, achieving food self-sufficiency, and ensuring food security at the family and national levels.

Traditional farm implements, unimproved crops and fertilizers, and inadequate animal breeds, as well as water use and availability, are important impediments to Ethiopian agricultural growth. The country's capacity to sustain agriculture through irrigation development has been limited [17].

The Legehida district is one of the most drought-prone and erratic rainfall regions. Due to this, the government is implementing different agricultural development programs to achieve livelihoods in rural farm households. Among these programs, small-scale irrigation development is primarily undertaken by the government, but due to economic scarcity, it is not widely implemented.

Irrigation is significant for improving agricultural production and food security. However, the farmers in the study area do not have enough knowledge to determine to what extent using irrigation is vital to boost agricultural production, household income, food security, and eradicate poverty; rather, they depend on rainfall. Accordingly, most rural people are highly vulnerable to poverty, socioeconomic problems, and food insecurity. As a result, in the district of Legehida where this study was conducted, in-depth comparative analysis studies are scarce, due to different factors such as irrigation technology, scheme and plot size, infrastructure, low income, limited extension access, and marketing opportunities. Therefore, the influence of small-scale irrigation on livelihood and determinant factors of household participation in the irrigation activity creates an empirical question in the study area. Hence, the study was conducted to reveal the seriousness of the problem and fill the gaps.

Thus, this study aimed to determine the factors affecting the participation of small-scale irrigation activities in rural farm households and to examine the influence of small-scale irrigation on household income and food security.

### 1.3. Research Questions

The study addressed the following research questions to achieve the above objectives related to the study on income, food security, and livelihood of rural farm households on different determinant factors.

1. What are the elements influencing farmer participation in small-scale irrigation?
2. How does irrigation affect food security significantly?
3. What are the main income sources for rural farm households?
4. To what degree do irrigation programs affect the livelihood of rural agricultural households?

### 1.4. Scope and Limitations of the Study

This study was undertaken to evaluate the influence of small-scale irrigation schemes on the livelihoods of rural farm households in the Legehida district. This research focuses on two small-scale irrigation schemes and the concept of livelihood is very broad; therefore, it is not possible to capture all aspects in a single study due to limitations imposed by time and financial resources and other related problems. Therefore, this study examines only the influence of small-scale irrigation schemes on users' income and food security. Furthermore, the information was acquired using a semistructured questionnaire survey, and the quality of the information was dependent on respondents' willingness, knowledge, and recall capacity. However, every attempt was made to collect valid data by convincing farm households of the study's aims.

### 1.5. Significance of the Study

The attainment of the abovementioned objectives is an essential instrument for agricultural growth; it increases the efficiency of small-scale irrigation and family food security. This study is noteworthy because it raises household awareness of the factors that determine small-scale irrigation involvement and its impact on food security. In general, the research's importance stems from its endeavour to provide actual information on the overall concerns of small-scale irrigation development in the study region, as well as its influence on increased income and better rural farm household livelihoods.

## 2. Research Methodology

### 2.1. Description of the Study Area

Legehida district is one of the rural districts of the Amhara National Regional State. It is in the northeastern part of the Amhara National Regional State, South Wollo Zone, Ethiopia, lying between 10° 49′ 48″ N latitude and between 39°22′ 12″ E longitude. The district has an altitude that ranges from 1644 to 3409 m.a.s.l. and an annual rainfall of 1250 mm with the minimum and maximum temperatures of 10 and 22, respectively. The total land area of the Woreda is estimated to be about 516,074 ha. Among these which is cultivated, 27607 ha, covered with forest 1320 ha, bush, and shrubs 30,725 ha, grazing 12,341 ha, and 2,992.02 ha of land in the district. Consequently, the district has a total population of 78845 that were included both male 39,182 and female 39,659, respectively.

People living in the study region rely mostly on agriculture for a living, and the area is noted for its low productivity owing to soil degradation. Crop and animal production systems are the primary source of income for the majority of the district's residents. Among the crops planted are teff, barley, wheat, bean, malt barley, finger millet, haricot bean, and chickpea. Different crops and fruits, such as tomato, potato, onion, lettuce, carrot, garlic, and apple, are grown in the region with irrigation during both the wet and dry seasons.

Irrigation was performed in the research region by various methods such as microdams (earth and concrete), river diversion, Acre ware dams, shallow wells, and ponds [2].

### 2.2. Data Source and Methods of Data Collection

The investigators should employ both primary and secondary data sources to appropriately meet the study goals. To capture all information on homes, primary data sources such as interviews, focus group discussions, and structured questionnaires with both open-ended and closed-ended questions might be employed. Secondary data sources would be gathered from various offices using either published or unpublished records. Agricultural agencies, environmental protection offices, and the district's Office of Water and Mineral Energy might all be included in a comprehensive assessment of the research.

#### 2.2.1. Primary Data

Structured surveys, key informant interviews, and focus group discussions were used to obtain primary data. Before data collection, all enumerators were instructed on data collection methodologies, survey content, and how to approach interviewees/best approach strategies.

The questionnaire also covered household wealth measurements such as household assets, livestock, and household agricultural production activities, as well as income quantities and sources. The same questionnaire was utilized for both irrigators and nonirrigators but with additional parts to cover particular irrigation-related topics.

#### 2.2.2. Focus Group Discussion

One of the qualitative data gathering approaches for this study was focus group discussions (FGD) with farmers. FGD obtained qualitative data through discussions with development agents, district agricultural and rural development office irrigation specialists, and irrigating and nonirrigating farmers. Each focus group consisted of nine people from the same village in the research region. FGD was used to create data that supplemented the structured questionnaire by explaining and addressing concerns raised by quantitative data.

#### 2.2.3. Key Informant Interview

Individuals in the research who were deemed competent and experienced in irrigation operations, rural livelihoods, and the socioeconomic situations of the community were selected and interviewed. Fifteen key informants were questioned, four of whom were extension officers and eleven of whom were farmers with knowledge of the scheme's farming operations.

### 2.3. Sample Size and Sampling Technique

Two small-scale irrigation (SSI) schemes, Berberti and Siba, were purposefully chosen based on population concentration in the schemes, the type of SSI they utilized, and the suggestions of the district's Agricultural and Rural Development Experts. A random sample of families should be drawn from each irrigation project and peasant organization. Household heads from the two kebeles with their different irrigation schemes were identified and classified into two groups: users and nonusers. A list of irrigating farmers was collected from scheme management, and farmers were stratified according to their position on the scheme to guarantee a fair representation of irrigation users. Using a simple random sampling approach, a sample of (113) irrigating farmers and (128) nonirrigators in the same geographic region were picked from these substrata (Table 1). Because the district is homogenous in terms of climate, resource endowment, and other criteria relevant to the research, this sample size is believed to reflect the population. The study used a simplified formula presented by Yamane [18], statistically calculated at the 95% confidence level, degree of variability = 0.05, and level of precision of 95%, to include the abovementioned sample household size.

(1) $$\begin{array}{c} n=\frac{N}{1+N{\left(e\right)}^{2}}, \end{array}$$

where *n* is the sample size, *N* is the population size (total household size), and *e* is the level of precision. Based on the number of total households in the sampling frame, the formula equated and reached a minimum of 241 respondents from the two selected kebeles.

### 2.4. Data Analysis Techniques

The data could be analyzed by using both descriptive and inferential statistical tools for the STATA software version 14 that would be used. The descriptive analyses are mean, median, and standard deviation, and the econometric data are analyzed using the propensity score matching technique.

### 2.5. Specification of the Model

#### 2.5.1. Propensity Score Matching (PSM) Methods

The propensity score matching method is a statistical technique used to estimate the causal effect of a treatment or intervention in observational studies. The study should be applied in a PSM technique, which has been widely applied as an impact evaluation instrument in the absence of baseline survey data for impact evaluation.

The justification for incorporating particular factors into the propensity score matching model is based on theoretical understanding, empirical data, and subject matter expertise. To improve the comparability of the treated and control groups, variables that are likely to affect both the treatment assignment and the result should be included. We want to reduce the possibility of confounding bias by balancing the distribution of variables between the groups through individual matching based on propensity scores.

To execute the PSM, one must complete five steps: estimate propensity scores, match treatment and control groups, verify common support conditions, assess matching quality, and perform sensitivity analysis [19]. However, we have combined the stages and provided them as follows for clarity and simplicity: the first step in the PSM technique is the estimation of propensity scores. There are two options to consider when estimating the propensity score. The first one relates to the estimated model and the second one to the variables that will be part of this model [19]. In terms of model selection, a probit/logit model has been used in several studies to evaluate impact analysis to calculate propensity scores [20]. Using a probit or logit model yields comparable findings when evaluating an individual's propensity score to be an adopter or nonadopter [21]. Nonetheless, the logit model was used in this work to estimate the propensity score of the studied homes because of its simplicity. Users of irrigation receive a value of 1, while nonusers receive a value of 0. The mathematical formulation of the logit model is as follows:

(2) $$\begin{array}{c} p_{i}=F\left(zi\right)=F\left(\alpha +\sum \beta_{i}x_{i}\right)=\frac{1}{1+{e^{-}}^{\left(\alpha +\Sigma \beta ixi\right)}}, \end{array}$$

where *e* is the base of the natural logarithm. Xi represents the ith independent variable. Pi is the probability that a household belongs to the participant given. *β*_*i*_ is the parameter to be estimated. (*Pi*) households will be participants (irrigators). (1-Pi) is the probability that a household will be nonparticipants (nonirrigators).

(3) $$\begin{array}{c} p_{i}=\frac{e^{zi}}{1+e^{zi}}, \end{array}$$

where Pi is the probability of participation in small-scale irrigation and 1-Pi is the probability of the household belonging to nonparticipants (nonirrigators) that is

(4) $$\begin{array}{c} \left(1-p_{i}\right)=\frac{1}{1+e^{z_{i}}},\\ \left(\frac{P_{i}}{1-p_{i}}\right)=\left(\frac{1+e^{z_{i}}}{1+e^{-z_{i}}}\right)=e^{z_{i}},\\ \left(\frac{p_{i}}{1-p_{i}}\right)=\left(\frac{1+e^{z_{i}}}{1+e^{-z_{i}}}\right)=e^{\left(\alpha +\Sigma \beta_{i}X_{i}\right)},\\ Zi=\ln\left(\frac{p_{i}}{1-p_{i}}\right)=\alpha +\beta_{1}X_{1}+\beta_{2}X_{2}\ldots \ldots \beta_{m}X_{m},\\ z_{i}=\alpha +\overset{m}{\underset{i=1}{\sum }}\beta_{i}\chi_{i}+\upsilon_{i}. \end{array}$$

In PSM, many matching algorithms may be employed to estimate the treatment impact on the treated. However, the most commonly used matching algorithms in PSM are nearest neighbour matching, radius matching, and kernel matching [22]. These matching approaches employ various ways of comparing the treated and control groups to ascertain the average effect of a specific participation irrigation activity or intervention.

### 2.6. Matching Estimators

The program evaluator's main objective after estimating the propensity scores is to find a suitable matching estimator. In theory, there exist various matching estimators. Only the most commonly used matching estimators are presented. They are as follows.

#### 2.6.1. Nearest Neighbour (NN) Matching

It is the simplest basic matching estimator. In NN matching, a person from a comparison group is chosen as a matching partner for a treated person who has the highest propensity score [23]. NN matching can be performed with or without replacement choices. In the case of NN matching with replacement, a comparison person can be matched to more than one treatment individual, resulting in higher match quality but lower estimate precision. Matching without replacement increases bias, although it may enhance estimate precision. Finding a suitable match via matching without replacement might be difficult in circumstances when the treatment and comparison units are highly diverse [23].

#### 2.6.2. Calliper Matching

As previously discussed, NN matching is vulnerable to poor matches when the nearest neighbour is far away. To solve this difficulty, the researcher employs a second alternative matching algorithm known as calliper matching. Caliendo and Kopeinig [19] define callipers matching as selecting a person from the comparison group as a matching partner for a treated individual who falls within a certain calliper (propensity score range) and is closest in terms of the propensity score. If the neighbourhood dimension is configured to be very tiny, likely, certain treated units will not be matched because the neighbourhood lacks a control unit. Conversely, the smaller the neighbourhood, the higher the quality of the matches [24]. One issue with calliper matching is determining which option for the tolerance level is the most realistic.

#### 2.6.3. Kernel Matching

This is another matching approach in which all treated units are matched with a weighted average of all controls, with weights inversely proportional to the distance between the treatment and control propensity scores [24]. Kernel weights each comparison group member's input, giving greater weight to those comparators that provide a better match. The most frequent strategy is to utilize the normal distribution (with a mean of zero) as a kernel, with the weight assigned to a specific comparator proportional to the frequency of the distribution for the difference in scores observed [25].

According to Caliendo and Kopeinig [19], one disadvantage of this technique is that potentially poor matches are employed because the estimator contains comparator observations for all treatment observations. As a result, accurate enforcement of the common support condition is critical for the kernel matching approach. One practical problem with its use is that it is not always evident how to establish tolerance. According to Mendola [26], 0.25 bandwidth kernel matching is the most commonly utilized.

The question remains as to how and which approach to use. Obviously, there is no single answer to this topic. The nature of the available dataset influences the selection of a matching estimator [25]. When the distribution of the propensity score between the comparison and treatment groups overlaps significantly, most matching algorithms produce comparable findings [22].

### 2.7. Region of Common Support Condition

**Figure 1:**
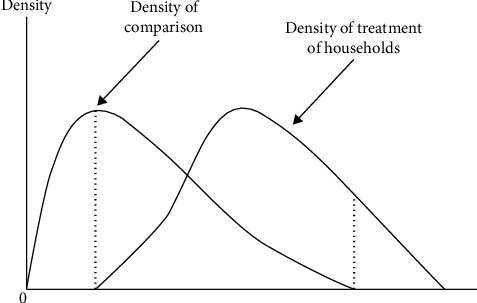
Region of common support condition. Source [27].

Figure 1 illustrates that by imposing a common support condition, any combination of features identified in the treatment group can also be observed in the control group [25]. The common support region is defined as the area containing the treatment and control group families' least and greatest propensity scores, respectively. It necessitates the deletion of all observations with propensity scores less than or more than the minimum and maximum of treatment and control, respectively [22].

### 2.8. Testing the Matching Quality

The balance test is a crucial consideration to make while doing PSM. While variations in variables are to be expected before matching, they should be avoided afterward. The PSM's principal function is to act as a balancing technique for covariates between the two groups. Propensity score estimation performance is thus measured by the resultant balance rather than the fit of the models used to generate the estimated propensity scores [28].

### 2.9. Variable Modification

#### 2.9.1. Dependent Variables

Participation in the small-scale irrigation system with families having access to irrigation and others without access to the irrigation scheme in the research region is the dependent variable of the first stage of this study.

#### 2.9.2. Outcome Variable

To evaluate the average treatment effect, food calorie intake per adult equivalent and annual income of rural farm households are proxy variables to examine the impacts of household food security and income.

Total annual household income: this variable is a continuous variable that represents the total annual household income in Ethiopian Birr. Evidence [29, 30] indicates that this variable has a favorable and significant impact on farmers' engagement in small-scale irrigation practices. Farmers are more likely to participate in small-scale irrigation activities if their overall family income is higher. This might be the case if farmers with greater incomes can easily pay irrigation costs compared to lower-income households. As a result, this characteristic was theorized to influence farmers' decision to participate in small-scale irrigation. The primary irrigation indicator reflects the amount of money earned (in cash) by the farmer or any household member from farm production, nonfarm revenue, and off-farm income. It is determined by the amount earned each year in birr from such sources.

Daily calorie intake per adult equivalent: daily calorie consumption per adult equivalent is an essential statistic for measuring family food security. Individual dietary intake may be assessed using a variety of approaches, including (i) 24-hour recall; (ii) food frequency surveys; and (iii) food records maintained by people or an observer. A reference period must be used in all dietary intake strategies. While some approaches rely on participant recollection (24-hour recall and food frequency questionnaire), others rely on the research participant, a proxy, or an observer noting meals as they are consumed. To assess family food security, both irrigation users and nonuser households provided information on the types and amounts of food consumed by their families in the 10 days preceding the survey. Following that, the total amount of food ingested was totalled and converted into calories (kcal) using the conversion factor of each food item consumed each day.

#### 2.9.3. Independent Variables

The independent variables hypothesized to influence household participation in small-scale irrigation are the combined effects of various factors such as physical, socioeconomic, and institutional factors explained as follows (Table 2) that have a significant impact on annual income and food (daily calorie intake per adult equivalent).

## 3. Results and Discussion

This chapter summarizes and examines the findings on the socioeconomic features of rural farm households, as well as the influence of small-scale irrigation on farmer livelihood. As a result of the descriptive analysis of chosen demographic and socioeconomic features of sample homes, the *t*-test is used to evaluate the significance of continuous variables, while the chi-square test is used to assess the significance of possible dummy variables (Appendix 8).

### 3.1. Age of Household Heads

The average age of the sample household heads was 44.16 years. The average ages of irrigation users and nonusers were 42.079 and 46 years, respectively (Table 3). In terms of age, the statistical analysis revealed a substantial difference between irrigation users and nonusers of homes. Dowd et al.'s [31] study stated that the age structure of a household is crucial for performing policies and engaging in tasks properly.

### 3.2. Family Size of Households

According to the survey results, the average family size of sampled irrigation users and nonirrigation users was 5.088 and 4.039, respectively (Table 3). However, the *t*-test results revealed that there was a significant mean difference between the two groups in terms of mean family size between irrigation users and nonirrigation users of the tested household. The findings revealed that the larger the family size, the more productive the workforce and the lower the labor cost for farm output.

### 3.3. Size of Cultivated Land Size

Land is well understood to be one of the most important components in manufacturing. As a result, it was projected that the likelihood of a family participating in irrigation activities would grow with farm size holding and decrease with nonirrigation users. According to the survey results, irrigation users owned more acreage than nonirrigation users, and this difference is statistically significant at a less than 1% probability level. As a result, the average total cultivated area for irrigation and nonirrigation users might be 1.221 hectares and 0.832 hectares, respectively (Table 3). As a result of this finding, total cultivated land sizes differed significantly between users and nonusers.

### 3.4. Total Livestock Unit

Farm animals play a significant part in rural livelihood. They provide draught power, protein supplements, status, income, animal dung for organic fertilizer, and transportation. It was quantified in terms of tropical livestock units, as in many previous similar research studies [32]. The combined mean of respondents' livestock holdings was 5.568 TLU. The mean comparison test scores for both users and nonusers were 6.647 and 4.615, respectively (Table 3). The comparison test results revealed a statistically significant difference in total livestock holdings between irrigation users and nonuser households.

### 3.5. Distance to Agricultural Office (FTC)

This is the distance in kilometers between the residential village and the nearest agricultural extension office. Households with access to agricultural extension services through development agents may have a choice of alternatives for adopting various contemporary and traditional technologies that may help them increase agricultural productivity. The average distance to the agricultural office/FTC was (1.741) and (2.027), respectively (Table 3). Similarly, the distance between agricultural offices//FTC makes a statistically significant difference between irrigation and nonirrigation users of rural farm households.

### 3.6. Sex of Household Head

Among the demographic factors, household head sex appeared to have a significant relationship with small-scale irrigation activity participation. The survey results revealed that the chi-square test was (12.704) and (pr < 0.0001), indicating that the gender of the household head is positively linked with the usage of small-scale irrigation. It is worth noting that, when all other variables are held constant, male-headed families are shown to be more likely than female-headed households to employ small-scale irrigation. This finding supports the premise that small-scale irrigation is appealing to male-headed families. This demonstrates that male farmers are more likely to participate in irrigation technology (adjusting for other variables in Table 4). Dillon [33] discovered, similar to this study, that the gender of the head is a variable that statistically and substantially explains involvement in irrigated agriculture.

### 3.7. Education Level of Household

It is commonly assumed that the education level of household heads has a significant impact on the adoption of irrigation technology and the improvement of agricultural output. Household heads with a higher education level were found to be more educated in irrigating homes. The chi-square test (39.430) found a significant connection between the education level and irrigation use at the 5% significance level. This suggests that household heads with more years of training are more likely to see the benefits of irrigation and to embrace and employ agricultural technologies more readily. As a result, farmers employ irrigation to boost their revenue and improve their food security. Access to quality education and skill development opportunities is critical for rural households to enhance their income-earning potential.

### 3.8. Off-Farm Job Participation of Households

The survey results indicated that 55.60% of the selected respondents had off-farm income involvement and 44.40% did not have off-farm income participation. In terms of irrigation access, 37% of irrigation users and 18.67% of nonusers had off-farm jobs on treated farms, while 10.30% of irrigation users and 34.02% of nonusers had no off-farm jobs on control. As a consequence, the chi-square test (42.760) and (pr < 0.0001) results demonstrated a substantial relationship between irrigation usage and access to off-farm income.

### 3.9. Soil Fertility Status

According to the survey results, the chi-square test of soil fertility 30.309 and (Pr < 0.0001) explains the positive association/influence on irrigation activity participation. Households with fertile land may generate a higher yield; moreover, if the households have access to irrigation, the production may grow significantly for both domestic use and sale. This condition motivates people to engage in small-scale irrigation, which increases income and improves food security for rural communities.

### 3.10. Training on Irrigation Farm

For the complete observation, about 57.26 percent of irrigation users received irrigation instruction, whereas approximately 42.74% of nonirrigation users received no irrigation training (Table 4). In terms of involvement in training on the chi-square result, there was a highly significant correlation between irrigation and nonirrigation users. This variable's outcome suggests that irrigation user families received more irrigation instruction than nonirrigation user households.

### 3.11. Access to Credit Service of Households

It is the primary source of money for impoverished farmers to acquire input and, ultimately, to embrace new technologies. Amhara Credit and Saving Institution (ACSI) and credit cooperatives are the primary sources of credit in the research region. According to the study results, 60.17% of the sample homes utilize credit. In terms of irrigation access, the survey results indicated that 37.34% of irrigation users and 22.82% of nonusers had used credit, while 9.95% of irrigation users and 29.87% of nonusers did not. Improving access to financial services, including microfinance, credit, insurance, and savings groups, can empower rural households and enable them to invest in income-generating activities. The chi-square test found a significant link between loan availability and irrigation use. This finding is controversial for Alemu [34]; there was no significant association between irrigation participation and access to credit services. The results of a research by Alexander et al. [35] indicated that many small businesses are likely to collapse in the absence of various forms of financial support.

#### 3.11.1. Household Income and Food Security

Respondents in the research region rely on agriculture for a living, work, money creation, food, and nonfood production and consumption. In the research region, agricultural revenue (irrigated and rain-fed crops) and off-farm and nonfarm income were the main sources of income (Appendix 6). As mentioned in the research region, the survey findings show that the relative percentage of revenues from grain to total yearly household income is the highest. As a result, grain cultivation is the primary source of revenue in the study region. It is followed by livestock production, off-farm production, and nonfarm production. Increased food calorie consumption and yearly birr income were substantially connected with better family food security status. The average daily calorie consumption of the entire sample homes was found to be 2266.2. According to the survey results, the average calorie consumption of irrigation user families and nonirrigation user households was 2408.85 and 1605.93 kilocalories per day, respectively (Table 5). The average yearly income of a household's irrigation and nonirrigation users was 29069.56 birr and 17110.53 birr, respectively (Table 5). As a result, the survey results revealed that irrigation user families were in a better position than nonirrigation user households in terms of income and daily calorie consumption for improving lifestyles.

### 3.12. Estimation Procedures of Econometric Models

The propensity score is estimated as the initial step in determining the treatment impact. To obtain these propensity scores for binary treatment cases, the logit or probit models can be utilized since both models often produce similar results when estimating the likelihood of involvement vs. nonparticipation [19].

The model was calculated using the propensity score matching approach developed by Leuven and Sianesi [36] using the STATA 14.0 computer program. Data from the two groups, namely, participant and nonparticipant homes, were pooled in the estimate so that the dependent variable has a value of 1 if the household has access to irrigation and 0 otherwise. The variance inflation factor (VIF) was used to determine if there was a strong multicollinearity problem among the explanatory variables. Before running the logit model to estimate propensity scores, the explanatory variables were tested for the presence of severe multicollinearity using several tests.

To discover the problem of multicollinearity among continuous explanatory variables, the variance inflation factor (VIF) approach was used. As a consequence, the VIF (Xi) result indicates that the data did not have a major problem with multicollinearity (Appendix 1). This is because the values of VIF for all continuous explanatory variables were significantly less than 10. Similarly, the contingency coefficients, which quantify the degree of connection between distinct discrete variables, were estimated to determine the degree of link among the discrete variables. The values of the contingency coefficient vary from 0 to 1, with zero indicating no correlation and values near 1 suggesting a significant degree of association. As a consequence, the calculation results indicated that there were no severe issues with correlations among discrete explanatory variables since the contingency coefficient did not surpass 0.75, which is commonly used as a cut-off point. As a result, all six discrete variables were included in the logistic analysis (Appendix 2). Furthermore, robust standard errors were used to overcome the problem of heteroscedasticity and normality.

### 3.13. Econometric Result

The logistic regression model revealed that seven variables, including land holding size, off/nonfarm income, education level, family size, dependency ratio, total livestock unit, and distance to nearest agricultural extension office/FTC, were significantly influenced by participation in small-scale irrigation at different statistically significant levels as shown in Table 6 and see also Appendix 4. The key explanatory variables that influence farm household irrigation participation are listed as follows.

#### 3.13.1. Household Head Education Level

According to the survey result, it is statistically significant at the 5% level and has a positive substantial influence on household food security. As a consequence, if the household was educated, involvement in irrigation activities increased by (0.2) percent; hence, educated families have a greater likelihood of adopting small-scale irrigation. Asayehegn et al. [37] discovered that education has a crucial impact on family decisions about technology adoption.

#### 3.13.2. Family Size

The survey results show that it was statistically significant at the 1% probability level, and households with a high family size utilize irrigation by (0.12) percent more. Similarly, the more people who participate in irrigation activities, the more crops are produced for consumption and sale, increasing revenue and improving food security. Farmers with larger family sizes were found to participate in small-scale irrigation practices more than those with smaller family sizes [38]. This might be the situation if family members are utilized as laborers in irrigated farming.

#### 3.13.3. Dependency Ratio

The survey results showed that involvement in small-scale irrigation was statistically significant at the 5% significance level. As a result of the variable's coefficient increasing at one unit, household involvement in irrigation activity increased by (0.077) percentage. As a consequence, the dependence ratio shows that the working population (active labor force, i.e., 15–64 year) actively participates in irrigation activities to create revenue or enhance food security. Similar findings suggest that the coefficient of the variable dependence ratio was statistically different from zero and found to be significant to impact the food security status of families [39].

#### 3.13.4. Cultivated Land Size

The survey results showed that it is significant at the 1% level and has a favorable association with irrigation participation. The data show that increasing the size of the cultivated area by one time improves the chance of becoming an irrigation user by (0.414) percentage. This is because the extent of farmed land encourages people to employ irrigation. The consequence is that the likelihood of being an irrigator increases with farm size (other factors remaining constant). According to the survey findings, when the area of cultivated land increases, a household's ability to vary the quantity and kind of the crop produced improves, which may lead to greater consumption and household food security. This finding is in line with Alemu [34] who showed a significant difference between irrigation-user households and nonuser households.

#### 3.13.5. Livestock Holding

It was favorably and statistically significant at the 1% significance level for irrigation activity participation. As a result, families have more animals, and irrigation usage has increased by (0.08) percent. Furthermore, having more livestock provides farm households with a greater opportunity to generate more cash through the sale of animals, which increases income and improves food security through direct consumption of livestock products.

#### 3.13.6. Distance to Farmers' Training Center

The model result indicates that this variable's coefficient has a negative association with irrigation participation, yet it is statistically significant at the 1% significance level. This suggests that families in the study region that are closer to the farmer's training center are more likely to participate in small-scale irrigation. As a consequence, the variable's coefficient was negative, and at 1 km of FTC, the chance of households participating in irrigation activities increased by (0.11) percent.

#### 3.13.7. Off-Farm Job Participation

At the 5% significance level, it had a favorable and significant effect on irrigation activity participation. As a result, farmers who engage in off-farm activities are less likely to participate in irrigation. Participation in off-farm activities may limit the allocation of manpower to farm operations. However, an increase of (0.05) percent in the marginal effect of this variable suggests that household heads who participate in off-farm work participation are more likely to participate in small-scale irrigation and contribute positively to revenue generation. Similar findings were obtained by [40]; however, Ayana [41] and Hadush [38] discovered that off-farm activities positively increase the amount of irrigation involvement.

#### 3.13.8. Propensity Scores

The logit model was used to predict the likelihood (propensity score) of each irrigation user (participant) and nonuser (nonparticipant) home based on observed household data (Table 7). The findings of the logit model of sample home participation in small-scale irrigation were used to generate propensity scores for the matching algorithms. The model employed thirteen (13) matching variables as explanatory variables, including the age of the household head, gender of household head, educational level of the household head, family size, dependency ratio, size of cultivable land, total livestock holding, household income in off-farm activities, distance to the nearest market, distance to the farmer training center, irrigation training, and access to credit service (Appendix 5). As a result, the dependent variable was a binary variable with a value of 1 for household involvement in small-scale irrigation and a value of 0 for nonparticipation.

**Figure 2:**
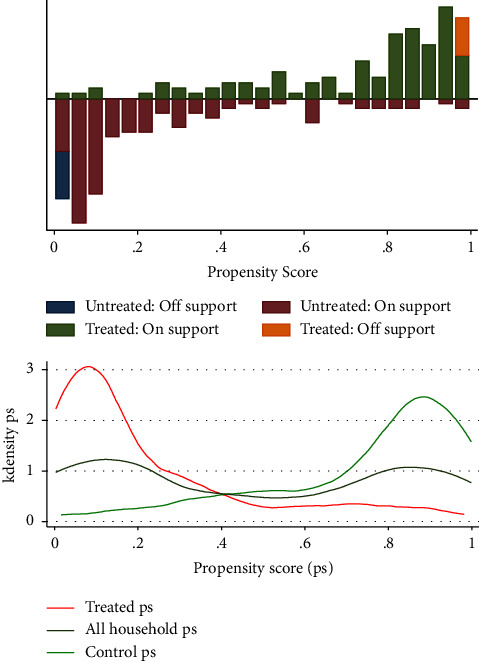
Prosperity score of treated, all households, and control.

Once the covariate propensity score estimation is complete, a common support area should be imposed on the propensity score distributions of both sample groups. Importantly, common support was increased by excluding treatment observations with estimated propensity scores larger than or less than the maximum or lowest of the comparison group propensity scores. Similarly, comparison group observations with propensity scores less than or greater than the treatment observations were dropped as shown in Figure 2.

#### 3.13.9. Schematic Presentation of the Common Support Condition

The predicted propensity scores for user (treatment) homes range between 0.0153 and 0.998 (mean = 0.744) and between 0.0028 and 0.981 (mean = 0.24) for nonuser (control) households, as shown in Table 7. The zone of shared support would thus be between 0.0153 and 0.9988. In other words, families with estimated propensity scores between 0.015 and 0.998 were excluded from the matching procedure. As a consequence, all of the treated families' approaches to 1 show that most irrigation users raise their income and enhance their food security more than the control groups (nonirrigation users).

#### 3.13.10. Matching Algorithm

To match the treatment and control groups in the common support region, several matching estimators were used. The final choice of a matching estimator was determined by several criteria, including the equal mean test, also known as the balancing test, pseudo-*R*^2^, and matching sample size. Specifically, a matching estimator that balances all explanatory variables (i.e., results in an insignificant mean difference between the two groups), a low pseudo-*R*^2^ value, and a large matched sample size are preferred. According to Table 8, the projected results of the test of the matching quality are based on the performance criteria mentioned above; hence, kernel matching with a bandwidth of (0.25) is the best estimator for the data in this matching result.

#### 3.13.11. Testing the Balance of Propensity Score and Covariates

After selecting the best performing matching algorithm, the balancing test is performed to determine the significance of the mean difference between all variables used for matching before and after matching [42]. The goal of the balancing test is to confirm that treatment is independent of unit characteristics after conditioning on observed characteristics (as estimated in the propensity score model), DX/P(X), where *X* is the set of characteristics that are thought to satisfy the conditional independence assumption.

**Figure 3:**
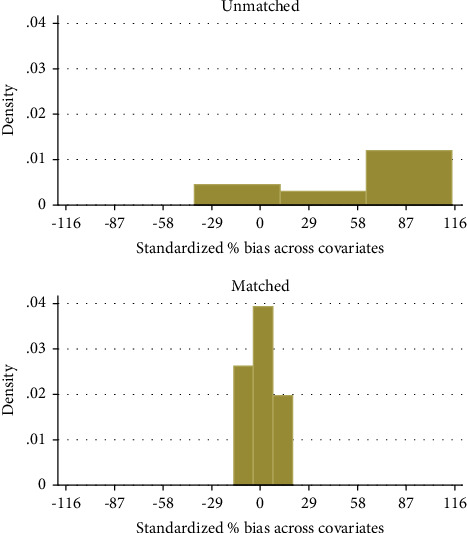
Propensity score and covariate balancing.

Any variation in the covariate mean between the two groups in the matched sampling has been erased after matching techniques, and hence, the pseudo-*R*^2^ should be low [19]. The basic goal of propensity score estimation is to balance the distributions of important variables in both groups, rather than to achieve a precise forecast of treatment selection. As indicated in Table 9, using the matching method technique resulted in a decrease in overall bias. Before matching, certain characteristics were substantially different for the two groups of respondents, including land holding size, off/nonfarm income, education level, family size, dependence ratio, total livestock unit, and distance to farmer training center (Table 9). However, following matching, these significant variables were conditioned to be insignificant, indicating that the disparities in the covariate mean between the treatment and control groups were abolished and balanced as shown in Figure 3.

#### 3.13.12. Estimating Treatment Effect on the Treated

These sections investigate the effects of small-scale irrigation on the end variables of family income and food security status before and after irrigation usage to achieve the study's main and specific stated objectives. Daily calorie intake per adult equivalent (DCAE) and annual income of rural families were evaluated as outcome variables.

Table 10 presents the estimation results and supportive evidence of the statistically significant effect of small-scale irrigation on the abovelisted outcome variables based on the already chosen matching algorithm. Accordingly, the small-scale intervention has resulted in a positive and statistically significant mean difference between irrigation users and nonirrigation users of farm households in terms of daily calorie intake per adult equivalent and annual income. A positive value of ATT confirmed that the income and food security have been improved due to the participation of irrigation activity in the study area.

After passing different steps of the matching technique, it was found that the use of small-scale irrigation increased the daily calorie intake and annual income of participant households by 244.162 kilocalories and 5234.258 ETB, birr, and this difference was significant at the 1% probability level, respectively (Appendix 3). Alemu [34] reported that the average increase in total calorie intake and consumption expenditure per adult equivalent among the participating families was 907.07 kcal and 2593.92 birr, respectively.

#### 3.13.13. Sensitivity Analysis

In this study, sensitivity analysis was carried out on the estimated average treatment effect using alternative matching estimators for both outcome variables and checked the observed characteristic of variables for both the treated and control groups (Appendix 7). Which were mentioned earlier, all matching estimators resulted in statistically significant effects on the households that use irrigation rather than nonirrigation users for the comparison of the outcome of household calorie intake and total annual income for the improvement of livelihoods.

## 4. Conclusion

This study investigates the impact of a small-scale irrigation system on farmer livelihoods in the Legehida area of South East Ethiopia. The research aims to reduce poverty and food insecurity and enhance income by identifying socioeconomic issues. Data were collected from primary and secondary sources using a simple random sampling approach and a quasiexperimental design. The logit and PSM models were applied to examine the influence of small-scale irrigation on farmer livelihoods. The study found that land holding sizes, off-farm income, education level, family size dependency ratio, total livestock unit, and distance to farmer training centers significantly influence farmers to practice small-scale irrigation and enhance food security in the area. The propensity score matching model revealed that the mean food intake of families on the treatment was 2388.3 Kcal per capita per day, higher than the national average. Access to small-scale irrigation increased the yearly income of irrigation user families by 5234.258 ETB compared with nonirrigation users.

In conclusion, access to small-scale irrigation is a feasible solution for securing household food needs in the study area. Encouraging rural farmers to use small-scale irrigation is critical for producing consumable food and marketable crops, reducing socioeconomic problems, and achieving sustainable livelihood improvement. The findings suggest improving water access for irrigation, raising farmers' awareness to enhance participation, and scaling up irrigation interventions to other irrigable land areas.

## Figures and Tables

**Table 1:** The target sample is taken from the population of each kebele.

Kebele	Total population	Total number of irrigation users	Total number of nonirrigation users	Total sample size population		
				Number of irrigation users	Number of nonirrigation users	Total sample
Siba	320	146	174	66	74	140
Berberti	290	106	184	47	54	101
Total	610	252	358	113	128	241

**Table 2:** Types of variable, measurement, and hypothesis expectation.

Types of variable	Description	Measurement	Hypothesis expectation
Dependent variable			
PSSI/participation in small-scale irrigation	Dummy, the status of participation in small-scale irrigation	1 = Participation on irrigation	+
Outcome variable			
Income	Continuous, income earned from irrigation production and livestock rearing	ETB	
Food security	Continuous, food calorie intake per day	AE	
Independent/explanatory variable			
AHH	Continuous, the age of household head of years old	Year	−
SHH	Dummy, gender of household head	1 = male 0 = otherwise	+
FSHH	Continuous, family size of the household	Number	+
DPRHH	Continuous, dependency ratio of household	Determine in age	+/−
EDSHH	Dummy, the educational status of a household	1 = Literate 0 = Illiterate	+
TCLHH	Continuous, total cultivated land holding size	Hectare	+
SFHH	Dummy, soil fertility status of land	1 = Fertile 0 = Otherwise	+
TLUHH	Continuous, total livestock holding size	TLU	+
EOFF	Dummy, engage in off-farm	1 = Participation in off-farm 0 = Otherwise	+
DMKTHH	Continuous, distance of market to household home	Km	−/+
DFTCHH	Continuous, distance of FTC to household home	Km	−
TRIIRHH	Dummy, training on the issue of irrigation	1 = Access to train and 0 = Otherwise	+
ACRSHH	Dummy, the level of access to credit service	1 = Yes 0 = Otherwise	+

**Table 3:** Descriptive statistics for continuous variables of the household characteristics.

Variables	Irrigation user		Nonirrigation user		Total		*t*-value
	Mean	Standard deviation	Mean	Stade	Mean	Stade	
AHH	42.079	9.629	46	10.274	44.161	10.147	−3.044^*∗*^
FASIZE	5.088	1.655	4.039	1.513	4.531	1.663	−5.140^*∗*^
DPRHH	4.747	1.507	4.559	1.472	4.647	1.489	−0.980
TLU	6.647	1.828	4.615	1.722	5.568	2.040	−8.876^*∗*^
SIZECUL	1.221	0.440	0.832	0.294	1.014	0.417	−8.147^*∗*^
DMKTHH	7.146	2.406	7.617	2.595	7.396	2.514	1.454
DFTCHH	1.741	0.970	2.027	1.300	1.893	1.163	1.917

The significance level at 1%, 5%, and 10% was expressed in asterisks ^*∗∗∗*^, ^*∗∗*^, and ^*∗*^, respectively.

**Table 4:** Descriptive statistics of dummy variables of household characteristics.

	0 = Otherwise	24	9.95	79	32.78	103	42.74	40.185
Access to credit service	1 = Yes	89	36.93	56	23.23	145	60.17	30.695
	0 = No	24	9.95	72	29.87	96	39.83	

Variable	Category of variables	Irrigation user		Nonirrigation user		Total		Chi2-test/*x*^2^
		Frequency	%	Frequency	%	Frequency	%	

Sex hhh	Male	96	39.83	83	34.44	179	74.27	12.704
	Female	17	7.05	45	18.67	62	25.73	

Education level hhh	1 = Literate	87	36.09	47	19.50	134	55.60	39.430
	0 = Illiterate	26	10.78	81	30.61	107	44.40	

Soil fertility land	1 = Fertile	88	36.51	55	22.82	143	59.34	30.309
	0 = Unfertile	25	10.37	73	30.29	98	40.66	

Off-farm	1 = Participate	88	36.51	46	19.08	134	55.60	42.760
	0 = Otherwise	25	10.37	82	34.02	107	44.40	

Training irrigation	1 = Access to train	89	36.93	49	20.33	138	57.26	

**Table 5:** Descriptive statistics for the outcome variable.

Variables	Irrigation user		Nonirrigation user		Total		*t*-value	*p* value
	Mean	S. d	Mean	S. d	Mean	S. d		
Annual income	29069.56	8313.035	17110.53	6302.97	22717.88	9507.46	−12.5^*∗*^	0.0001
Daily ADE	2408.85	422.01	1605.93	534.51	1982.40	628.89	−12.8^*∗*^	0.0001

The significance level at 1%, 5%, and 10% was expressed in asterisks ^*∗∗∗*^, ^*∗∗*^, and ^*∗*^, respectively.

**Table 6:** Results of logistic regression.

Variables	Coefficient	Std. err	*z*-value	*p* value	Marginal effect dy/dx
SEXHHH	0.6988549	0.4662645	1.50	0.134	0.1662
AGEHHH	−0.0035608	0.0206931	−0.17	0.863	0.0008
EDSHHH	0.8521269	0.4223542	2.02	0.044^*∗∗*^	0.2059
DPRHH	0.5631012	0.1586323	−1.98	0.047^*∗∗*^	0.0775
FASIZE |	0.5631012	0.1513425	3.72	0.001^*∗*^	0.1388
SIZECUL	1.681016	0.6226108	2.70	0.007^*∗*^	0.4145
SFLAND	0.23163	0.1513425	0.54	0.591	0.0569
TLU	0.3496817	0.430924	2.91	0.004^*∗*^	0.0862
EOFF	0.8273996	0.4230811	1.96	0.051^*∗∗∗*^	0.2001
DMKTHH	−0.0091119	0.0806091	−0.11	0.910	0.0022
TRAIRR	0.4589877	0.4232527	−2.57	0.278	0.1122
DFTCHH	−0.4542078	0.1769482	−2.57	0.010^*∗*^	0.1120
ACRSHH	0.3397416	0.4567797	0.74	0.457	0.0832
Constant	−5.949574	1.551089	−3.84	0.001	
Number of observations	=241				
Prob > chi2	=0.0001				
LR chi2 (13)	=145.44				
Pseudo−*R*^2^	=0.4365				

The survey result shows ^*∗∗∗*^, ^*∗∗*^, and ^*∗*^ level of significance at 1%, 5%, and 10%, respectively.

**Table 7:** Schematic representation common support region.

Variables	Observation	Mean	Standard deviation	Min	Max
Treated	113	0.7440055	0.2422463	0.0153	0.998
Control	131	0.248	0.225	0.0028	0.981
All	244	0.468	0.356	0.0028	0.998

**Table 8:** Performance of matching estimators.

Matching algorism	Bandwidth pseudo-	pseudo − *R*^2^	Insignificant variable	Matching sample size
Radius calliper (RC)	Radius (0.01)	0.078	12	111
	Radius (0.05)	0.042	10	217
	Radius (0.1)	0.035	11	224
	Radius (0.25)	0.039	12	224

Nearest neighbour (NN)	NN (1)	0.08	9	224
	NN (2)	0.058	11	224
	NN (3)	0.055	10	224
	NN (4)	0.045	10	224

Kernel matching (KM)	Bw (0.01)	0.067	12	111
	Bw (0.1)	0.036	11	224
	Bw (0.25)	0.031	13	224
	Bw (0.5)	0.061	10	224

Source: own econometric result (2021).

**Table 9:** Propensity score and covariate balancing.

Variables	Sample	Mean		Reduction bias (%)	*T*-test	
		Treated	Control		*t*-value	*p* value
Sexhhh	U	0.84956	0.64844		3.65^*∗∗∗*^	0.001
	M	0.84906	0.80573	78.5	0.83	0.406

Agehhh	U	42.08	46		−3.04^*∗∗∗*^	0.003
	M	42.292	40.901	64.5	1.19	0.234

Edshhh	U	0.76991	0.36719		6.84^*∗∗∗*^	0.001
	M	0.75472	0.78374	92.8	−0.5	0.618

Dprhh	U	4.7479	4.5595		0.98	0.328
	M	4.7812	5.0177	−25.5	−1.15	0.252

Fasize	U	5.0885	4.0391		5.14^*∗∗∗*^	0.001
	M	5.0472	5.0167	97.1	0.13	0.893

Sizecul	U	1.2212	0.83203		8.15^*∗∗∗*^	0.001
	M	1.184	1.1117	81.4	1.29	0.197

FPSFland	U	0.77876	0.42969		5.86^*∗∗∗*^	0.001
	M	0.77358	0.79442	94	−0.37	0.714

Tlu	U	6.6473	4.6154		8.88^*∗∗∗*^	0.001
	M	6.3906	6.2694	94	0.54	0.589

Eoff-farm	U	0.77876	0.35938		7.18	0.001
	M	0.77358	0.79159	95.7	−0.32	0.752

Dmkthh	U	7.146	7.6172		−1.45	0.147
	M	7.217	7.1161	78.6	0.27	0.785

Traing irri	U	0.78761	0.38281		6.92^*∗∗∗*^	0.001
	M	0.77358	0.75329	95	0.35	0.73

Dftchh	U	1.7409	2.0273		−1.92^*∗∗*^	0.056
	M	1.7426	1.7125	89.5	0.23	0.82

Accredit	U	0.78761	0.4375		5.91^*∗∗∗*^	0.001
	M	0.77358	0.83819	81.5	−1.19	0.236

^*∗∗∗*^, ^*∗∗*^, and ^*∗*^ shows the level of significance at 1%, 5%, and 10%, respectively. Source: own econometric result (2021).

**Table 10:** The average treatment effect on the treated.

Variables	Mean		ATT	Se	*t*-stat
	Treated	Control			
Income	28423.2075	23188.9491	5234.25845	1527.21004	3.43
Daily calorie/ADE	2388.30189	2144.14014	244.161748	116.621446	2.09

## Data Availability

The collected and analyzed data during the current study are available upon reasonable request from the corresponding author.

## Supplementary Materials

Supplementary Materials Appendix 1: Livestock conversion factor. Appendix 2: Conversion factor for adult equivalent. Appendix 3: Food poverty line /conversion factor for each food item. Appendix 4: Results of the logistic model. Appendix 5: Result regressions. Appendix 6: Variance inflation factor. Appendix 7: Sensitivity analysis result. Appendix 8: Shortened version of survey questionnaires.
