# Supplementary material for: Influence of Small-Scale Irrigation on Livelihoods of Rural Farm Households in the Case of Legehida District, Ethiopia
Source: ScientificWorldJournal. 2024 May 23;2024:9982796. doi: 10.1155/2024/9982796 (PMC11139502; doi:10.1155/2024/9982796)
Supplement: Supplementary Materials — Appendix 1: Livestock conversion factor. Appendix 2: Conversion factor for adult equivalent. Appendix 3: Food poverty line /conversion factor for each food item. Appendix 4: Results of the logistic model. Appendix 5: Result regressions. Appendix 6: Variance inflation factor. Appendix 7: Sensitivity analysis result. Appendix 8: Shortened version of survey questionnaires. [file 9982796.f1.pdf]

APPENDIX

Appendix 1: Livestock conversion factor

Type of livestock	Conversion factor
**Cow/ox**	1.00
**Young bull**	0.80
**Heifer**	0.75
**Calf**	0.20
**Weaned calf**	0.34
**Horse/mule**	1.10
**Sheep/got young**	0.13
**Sheep/got adult**	0.06
**Donkey adult**	0.70
**Donkey young**	0.35
**Chicken**	0.013

Source: Freeman, *et al*. 1996

Appendix 2: Conversion factor for adult equivalent

AGE	Sex	ADE[1]
**0-7**	Male	0.6
	Female	0.6
**8-10**	Male	0.8
	Female	0.8
**11.15**	Male	0.9
	Female	0.9
**16-24**	Male	1.0
	Female	0.8
**25-50**	Male	1.0
	Female	0.8
**51-65**	Male	0.9
	Female	0.7
**>65**	Male	0.8
	Female	0.6

AE = Adult equivalent

Source: Stork, *et al;* 1991

Appendix 3: Food poverty line /conversion factor for each food item

Food item	Average consumption per day (kg)	Conversion factor of food item	Calorie per day from average consumption (c.f*kg)
Teff	0.421	3589	1510.969
Wheat	0.161	3623	583.303
Sorghum	0.086	3805	327.23
Barley	0.012	3723	44.676
Bean	0.026	3514	91.364
Peas	0.031	3553	110.143
Lentils	0.0069	3522	24.301
Paper	0.012	225	2.7
Onion	0.16	713	114.08
Potato	0.009	8970	80.73
Oil	0.015	8970	134.46
Tomato	0.008	707	5.656
Cabbage	0.07	205	14.35
Coffee	0.068	1103	75.004
Milk	0.19	7370	1400.3
Sugar	0.132	3850	508.2
Total	1.4079	57442	96299.02

Appendix 4: Results of logistic model

```
Iteration 0:   log likelihood = -166.58136
Iteration 1:   log likelihood = -95.057587
Iteration 2:   log likelihood = -93.883742
Iteration 3:   log likelihood = -93.862773
Iteration 4:   log likelihood = -93.862765

Logistic regression                               Number of obs     =        241
                                                  LR chi2(13)       =     145.44
                                                  Prob > chi2       =     0.0000
Log likelihood = -93.862765                       Pseudo R2         =     0.4365
```

pssir	Coef.	Std. Err.	z	P>|z|	[95% Conf.	Interval]
sexhhh	.6988549	.4662645	1.50	0.134	-.2150068	1.612717
agehhh	-.0035608	.0206931	-0.17	0.863	-.0441185	.0369969
edshhh	.8521269	.4223542	2.02	0.044	.024328	1.679926
dprhh	-.314556	.1586323	-1.98	0.047	-.6254697	-.0036423
fasize	.5631012	.1513425	3.72	0.000	.2664754	.8597271
sizecul	1.681016	.6226108	2.70	0.007	.4607214	2.901311
fpsfland	.23163	.430924	0.54	0.591	-.6129655	1.076226
tlu	.3496817	.11998	2.91	0.004	.1145251	.5848382
eoff	.8273996	.4230811	1.96	0.051	-.0018242	1.656623
dmkthh	-.0091119	.0806091	-0.11	0.910	-.1671029	.1488791
trairr	.4589877	.4232527	1.08	0.278	-.3705723	1.288548
dftchh	-.4542078	.1769482	-2.57	0.010	-.8010198	-.1073958
acrshh	.3397416	.4567797	0.74	0.457	-.5555301	1.235013
_cons	-5.949574	1.551089	-3.84	0.000	-8.989652	-2.909497

Appendix 5: Result regressions

```
. reg pssir sexhhh agehhh edshhh dprhh fasize sizecul fpsfland tlu eoff dmkthh trairr dftchh acrshh
```

Source	SS	df	MS			
				Number of obs	=	241
				F(13, 227)	=	15.84
Model	28.550383	13	2.19618331	Prob > F	=	0.0000
Residual	31.4662145	227	.138617685	R-squared	=	0.4757
				Adj R-squared	=	0.4457
Total	60.0165975	240	.250069156	Root MSE	=	.37231

pssir	Coef.	Std. Err.	t	P>|t|	[95% Conf.	Interval]
sexhhh	.0806497	.0587282	1.37	0.171	-.0350724	.1963717
agehhh	-.0011804	.0026821	-0.44	0.660	-.0064655	.0041046
edshhh	.1297637	.0578671	2.24	0.026	.0157383	.243789
dprhh	-.0378111	.0191403	-1.98	0.049	-.0755264	-.0000957
fasize	.0774165	.0174029	4.45	0.000	.0431246	.1117083
sizecul	.229453	.0693102	3.31	0.001	.0928795	.3660266
fpsfland	.061439	.0578402	1.06	0.289	-.0525334	.1754114
tlu	.0461012	.0145546	3.17	0.002	.0174218	.0747805
eoff	.1391817	.058083	2.40	0.017	.024731	.2536324
dmkthh	.0011779	.0102759	0.11	0.909	-.0190705	.0214263
trairr	.0721538	.0597518	1.21	0.228	-.0455854	.189893
dftchh	-.0603982	.0212042	-2.85	0.005	-.1021806	-.0186159
acrshh	.0563654	.0624434	0.90	0.368	-.0666773	.1794082
_cons	-.3590175	.1825544	-1.97	0.050	-.7187353	.0007004

.

Appendix 6: Variance inflation factor

Variable	VIF	1/VIF
acrshh	1.62	0.615494
tlu	1.53	0.654780
trairr	1.52	0.658289
fasize	1.45	0.689282
sizecul	1.45	0.689918
eoff	1.45	0.690636
edshhh	1.44	0.695799
dprhh	1.41	0.711064
fpsfland	1.40	0.712547
agehhh	1.28	0.779662
dmkthh	1.16	0.864831
sexhhh	1.15	0.872767
dftchh	1.05	0.948665
Mean VIF	1.38	

Appendix 7: Sensitivity analysis result

Income						
Gamma	sig+	sig-	t-hat+	t-hat-	CI+	CI-
1	1.60E-07	1.60E-07	4700	4700	3000	6450
1.1	1.40E-06	1.50E-08	4312.5	5090	2577.5	6867.5
1.2	8.10E-06	1.40E-09	3987.5	5405	2250	7230
1.3	0.000035	1.20E-10	3693.38	5685	1987.5	7550
1.4	0.000122	1.10E-11	3430	5987.5	1715	7890
1.5	0.000351	9.80E-13	3190	6300	1392.5	8150
1.6	0.000875	8.60E-14	2925	6537.5	1081.25	8467.5
1.7	0.00193	7.50E-15	2662.5	6775	800	8774.62
1.8	0.003846	6.70E-16	2425	7017.5	600	9005
1.9	0.007039	1.10E-16	2237.5	7232.5	437.5	9217.5
2	0.011982	0	2075	7412.13	262.5	9512.5

Kalorie in take					
sig+	sig-	t-hat+	t-hat-	CI+	CI-
7.40E-08	7.40E-08	229.541	229.541	147.709	360.66
6.60E-07	6.40E-09	205.179	268.851	135.749	382.31
4.10E-06	5.50E-10	186.551	300.938	125.63	403.742
0.000019	4.70E-11	171.577	321.38	117.044	421.565
0.000067	3.90E-12	158.973	335.939	107.217	432.31
0.0002	3.30E-13	152.007	352.688	97.3996	440.802
0.000515	2.70E-14	145.021	366.165	88.9464	449.047
0.001172	2.20E-15	137.683	378.774	81.2186	457.791
0.002402	2.20E-16	130.788	390.06	70.9183	464.63
0.004513	0	125.572	404.224	53.8257	471.427
0.007871	0	119.961	415.141	38.3164	478.75

Appendix 8: Shortened version of survey questionnaires

Enumeration

Enumerator's Name	Interview Date	Time Int. Started	Time Int.	Interview Place	Checking (√)	Signature

Part I; Control Section: Demographic and socio-economic characteristics of respondents

1. Questionnaire identification number ________________

2. Date of interview (Ethiopian calendar) ______________________

3. Name of respondent's Kebele: 1) Siba 2) Berberti

4. Name of respondent's village/Got/ _______________________

5. Sex of household head: (1) Male (2) female.

6. Age of household head: __________________.

7. Family size of household? Male--------- Female-------------- Total------------

8. Could you list out the age category of your household members?

1) 0-5 years --------- 2) 6-17 years ---------- 3) 18- 65 years ------- 4) 65 and above --------

9. Religion of household: 1) Muslim 2) Orthodox 3) other (specify)_______________

10. Marital status of household: (1) Married (2) Single (3) Divorced (4) Widowed

11. Educational level of household head (Years of schooling): (1) literate (2) illiterate, if the household is literate describe the grade---------------------

12. Social status or responsibility in your community: (1) Kebele Administration (2) Religious leader (3) Local institution/CBOs (4) Traditional healer 5) Elder (6) Agricultural cooperatives administration (7) Water committee (8) others (specify)_________________

Part II: Access to irrigation and improved agricultural practice

1. Are you an irrigation user in small scale irrigation: 1) yes 0) No

2. Which small-scale irrigation type do you use? 1) Modern micro dam 2) traditional river diversion 3) motor pump 4) treadle pump 5) others specify (if any), ------------------

3. Have you ever experienced in irrigation activity? 1) Yes 0) No

4. If yes, for how long you have been practicing irrigation activity? ---------------- Years.

5. Have you cultivated your irrigable plot in full scale? 1) Yes 0) no

6. If no, write the most important inhibiting factors in the following table

Factor	Rank	What is your response
Shortage of water		
Labor		
Scarcity of input		
Credit		

7. How many times you produce per year on irrigable land? 1) Once 2) twice 3) three times 4) four times

8. If you are not using small scale irrigation schemes, what are the main reasons for not using? 1)Shortage of land for irrigation 2) lack of awareness about irrigation 3) production input problems 4) lack of oxen 5) problem of sufficient irrigation water

9. Have you used irrigation planning? 1) Yes 0) no

10. If yes, what criteria you used to decide when and type of irrigated crops? 1) Price of the crop 2) cost of production 3) cash income from the sale of the crop

11. Do you have access to irrigation water? 1) Yes 0) no

12. If yes, what is the main source of your irrigation water? 1) Hole 2) river 3) lake 4) well 5) others

13. How far is your irrigation plot from water source? ------------------------km (hour)

How far is the nearest farm land? ------------------------km (hour)

The furthest farm land ------------------------km (hour)

14. Do you use any of improved agronomic practices? 1) Yes 0) no

15. If say, yes how many times do you use improved agronomic practices? 1) Always 2) sometimes 3) often 4) others

16. Total area of land cultivated during last year on which fertilizer was used_______(in tsimad)

17. Total area of land covered by improved seeds during the last one year___________ (in tsimad).

Justify the activity given in the table below

No	Activity	yes	no	Unit	amount	Source	Value in birr
1	Did you purchase any inorganic fertilizer/DAP&UREA/ for use on your field?						
2	Did you use any manure/compost from your herd on your field?						
3	Did you purchase improved seeds for use on your field?						
4							
5							

Part III: Asset profile of respondents

1. How many hectares of land do you have? 1) Below 0.5 ha 2) 0.5-1 ha 3)1-2 ha 4) above 2ha
2. What type of your soil quality? 1) Fertile 0) others
3. How much of your land is used by irrigation? ______________ (hectares).
4. Have you cultivated the total of your irrigable land during the last crop production season? 1) Yes 0) no
5. Have your own active family members (11 years and above) participated in farm activity? 1) Yes 0) no
6. If yes, specify the number of the family members engaged in the farm activity: _________
7. What type of activity they are engaged in? 1) Weeding 2) harvesting 3) threshing 4) watering 5) planting 6) ploughing
8. What are the main sources of the labour for your irrigation activity? 1) Family labour 2) hired labor
9. Did you face any labor shortage during the last production season? 1) Yes 0) No

10. If yes, how did you solve the labor shortage? 1) Through hiring additional daily labourer's 2) through debo/jigi 3) using family labors

11. Can you easily get labor to hire when you are in need? 1) Yes 0) No

13. Do you have any livestock? 1) Yes 0) no

14. If yes, indicate number and types of livestock you owned currently in the following

Name of livestock	Number owned and presented on your farm	If you would sell how much you received in the last year.	Total value in birr
Oxen			
Cow			
Heifer			
Bull			
Calf			
Goat			
Sheep			
Mule			
Horse			
Chicken			
Beehives			
Camel			
Donkey			
Other specify			

Part IV: Food security and income source

1. How many mealtimes does your household consume basic food on average in a day?

1) One time in a day 2) two times in a day

3) Three times in a day 4) more than three times in a day

2. Do you tell us the consumption of each of the food items in your family for the current year?

Food items consumed	Unit	Quantity in kg	Unit market	Total value	1. own 2. gift 3. purchased 4. loan 5. aid
Teff					
Maize					
Sorghum					
Wheat					
Barely					
Peas					
Beans					
Chickpea					
Millet					
Lentils					
Milk					
butter					
Egg					
Coffee					
Honey					
Sugar					
Oil					
Pepper					
Bread					
Enjera					
Cabbage					
Onions					
Tomato					

3. Estimation of household incomes from on farm and off-farm activities for the last one year?

Item	Quantity sold in the year	Value earned from sales of indivdual(in birr)	Total Value earned from sales (in birr)

Livestock			
Crop			
Vegetables and fruit			
Others			

4. Did you participate in non-farm activities? 1) Yes 0) No

5. If your answer to Q#3 is "Yes", how much did you receive as income from your participation?

s/n	Activities(participation)	Value in (birr) in the last year
1	Petty trade	
2	Handcrafts	
3	Livestock and livestock output trade	
4	Sale of local drinks	
5	Construction labor	
6	Weaving	
7		
8		
9		
10		

Part V: Agricultural Extension, marketing access &credit institution

1. Where do you obtain extension services? 1) Development agent 2) farmers group 3) FTC 4) radio 5) NGO 6) district agricultural office

2. Have you ever been visited by agricultural development agents? 1) Yes 0) no

3. If yes, how many times did they contact you? 1) Twice a week 2) once a week 3) monthly 4) seasonally

4. Did you practically use any of the advice on your farm land during last production season? 1) Yes 0) no

5. If no, why didn't you use it? 1) Irrelevant 2) not timely 3) lack of finance to afford

6. Is there farmers training centres (FTC) in your Tabia? 1) Yes 0). No

7. How far is the FTC from your home ________________ in Km?

8. How long do you take from your home to FTC______________ in minutes?

10. If yes, in which topics you had been trained from the lists mentioned in the table below (see codes listed under the table).

Training topics	How many Rounds of training you have been taken	For how long you taken (days)of training

1=livestock production 2= fruits and vegetables 3=crop diversification 4=marketing 5=irrigation 6=post-harvest processing 7= storage of farm produce 8=farm management 9= credit 10= household Livelihood ……11=others

11. Did you get market information about prices and conditions of agricultural inputs and out puts?

1) Yes 0) No

12. If yes, what is the source information? 1) Radio 2) Television 3) Newspaper 4) Mobile 5) Others specify________________

13. Where did you sell your product? 1) At village market 2) A district market 3) At regional market,4) At national market 5) others (specify) ______________________________

14. What is the distance of your residence from the market ___________________ (in Km)?

15. What means of transport do you use to transport your product to the market?

1) Vehicles 2) Animal labor 3) Human labor 4) Other (specify)__________________________

16. Did you get fair price for your product at this particular time? 1) Yes 2) No

17. If no, what are the reasons? 1) No demand 2) More supply 3) Others (specify)_______________

18. Why did you sell at that particular time? 1) To appropriate family requirements 2) To pay debts 3) Other_________

19. Had you receive any credit in the past one year? 1) Yes 2) No

20. If yes, for what purpose (s)? 1) Purchase of seeds 2) Purchase of fertilizer 3) purchase of oxen

4) For family consumption 5) Others (specify)______________________

21. When do you usually take the credit? ___________________ (months)

22. What are the Sources of credit? 1) Credit institutions 2) Commercial banks 3) Friends 4) Other__________

23. If not why? 1) Lack of access to credit 2) No need for credit 3) High interest rate 4) Others---

Checklists for Focus Group Discussion (FGD) and Key informant Interview

1. What are the major types of farming activities practiced by smallholders in the area?
2. How do you see the function of the constructed irrigation scheme in availing of enough food for target community?
3. What are the major challenges observed underutilizing the small-scale irrigation schemes?

4. What are the major determinant factors for producing enough food at household level?

5. What are the major crop types you cultivate that assure rural farm household livelihoods?

6. What is the influence of access to irrigation on crop production and employment opportunities for rural household in the area?

7. What are the sources of irrigation water?

8. What is the role and number of irrigation scheme water use association?

9. What are the rules and regulation (bylaws) practiced by the irrigation scheme water association?

10. What are the major constraints or draw backs of to use irrigation water in order of importance?

11. What is the nature of conflict related to irrigation water and measures taken to resolve conflicts?

12. Is the irrigation water available throughout the year?
